# The importance of genetic research in cases of severe male factor infertility: A case of 46,XX testicular disorder of sex development

**DOI:** 10.5935/1518-0557.20210092

**Published:** 2022

**Authors:** Dalana Faleiro, Betina Iser, André Anjos da Silva, Marcos Alexandre Höher

**Affiliations:** 1 Hospital Bruno Born (Centro de Reprodução Humana Bruno Born); 2 Programa de Pós-Graduação em Ciências da Saúde-Ginecologia e Obstetrícia, Universidade Federal do Rio Grande do Sul (UFRGS), Porto Alegre, Brazil; 3 Programa de Pós-Graduação em Ciências Médicas, Universidade do Vale do Taquari - Univates, Lajeado, Brazil

**Keywords:** 46,XX male, de *la Chapelle* syndrome, SRY gene, male infertility, case report

## Abstract

46,XX testicular disorder of sex development is a rare syndrome characterized by an inconsistency between genotype and phenotype. Affected individuals present variant genitalia between male and ambiguous, non-functional testicles, non-obstructive azoospermia, generally accompanied by hypergonadotropic hypogonadism, a condition known for high levels of gonadotrophic hormones. In some cases, disorders of sexual development are diagnosed during puberty. However, a significant number of individuals show physical characteristics common to males that are not clinically suspicious. As a result, patients with the condition may remain undiagnosed. Many individuals with the condition are diagnosed as adults, due to infertility. The present study discusses the case of an individual who underwent karyotyping for sterility and was found to be a 46,XX male. Despite having a female karyotype, the presence of the sex-determining region Y gene explains the manifestation of masculine secondary sex characteristics. This report highlights the importance of genetic evaluation, considering that carriers may present significant complications resulting from the disorder. Based on correct diagnosis, it is possible to improve a carrier's quality of life through multidisciplinary approaches and help them achieve pregnancy through assisted reproductive technology treatments.

## INTRODUCTION

Infertility is a health issue that affects 15-20% of couples of reproductive age characterized by the inability to achieve pregnancy after 12 months of having sex without the use of contraceptive methods (Datta *et al*., 2016; Gilany *et al*., 2015; Szczykutowicz *et al*., 2019). Male factor infertility accounts for 20-60% of the cases, appearing in isolation or in association with female infertility (Szczykutowicz *et al*., 2019; Alahmar, 2019; Nardelli *et al*., 2014; Ferlin *et al*., 2007). The substantial involvement of males in cases of conjugal infertility demonstrates the importance of assessing the male reproductive system and the conditions that influence reproductive capacity, including functions related to the endocrine system, spermatogenesis, genetic changes and processes involved in fertilization (Halder *et al*., 2017). Azoospermia, frequently seen in daily clinical practice and characterized by the complete absence of sperm in the ejaculate, accounts for 10-15% of male infertility cases (Katz *et al*., 2017; Hamada *et al*., 2013; Krausz & Riera-Escamilla, 2018). Genetic changes related to chromosomal abnormalities have been found in 15% of males with non-obstructive azoospermia (Krausz & Riera-Escamilla, 2018).

46,XX testicular disorder of sexual development (DSD), also known as de la Chapelle syndrome, is a rare condition (1 in 20,000-30,000 male individuals) in which a discrepancy exists between the phenotype and genotype of an individual (Gilany *et al*., 2015; Krausz & Riera-Escamilla, 2018; De La Chapelle et al., 1964; Mohammadpour Lashkari *et al*., 2016). The main characteristics of individuals with 46,XX testicular DSD are having male external genitalia and azoospermia. Patients with the condition may also present with cognitive problems, sexual dysfunction, and reduced hair distribution (Bianco *et al*., 2011; Délot & Vilain, 2015). Considering the importance of diagnosis and the repercussions of the condition in the affected individual's life, it is crucial to report cases to the scientific and medical communities in order to improve diagnosis, treat, and follow patients with the condition. This paper reports the case of an individual with 46,XX testicular DSD diagnosed at a center for human reproduction (CRH) in Southern Brazil.

## PATIENT INFORMATION AND DISCUSSION

A couple suffering with infertility for two years went to a CRH in Southern Brazil. Although the male partner in the couple had azoospermia, the causes of his condition had not been investigated and the couple was unaware of the chromosomal alteration that he had. The Research Ethics Committee approved the study (Plataforma Brasil certificate no. 45463021500005310) and the patient gave consent to having the case published.

The patient was a 183-cm tall male Caucasian weighing 100 Kg with a phenotype that is typically associated with having a eunuchoid body habitus, i.e., increased atypical fat distribution and reduced virilization. Individuals with de la Chapelle syndrome have both female and male physical characteristics, with greater amounts of fatty body mass in relation to lean body mass (Damiani et al., 2005; Majzoub *et al*., 2017), as observed in the individual described in this case report.

The patient in question had a history of surgical correction of gynecomastia performed at the age of 27. Physical examination revealed he had a small penis and reduced pubic hair. The urinary system showed no abnormalities. The patient had testicular alterations, and ultrasound examination was performed in other areas of the scrotal pouch to check for possible anatomical lesions and malformations. Both testicles were atrophic, heterogeneous, and considerably reduced in volume. The echogenic areas within them were suggestive of fibrosis. The right testicle was located in the inguinal canal (cryptorchidism), measuring 1.4 x 1.0 x 0.9 cm, with a volume of 0.7 mL. The left testicle was located in the scrotum, measuring 1.3 x 1.0 x 1.0 cm, with a volume of 0.7 mL. The structures of the epididymides did not show any noticeable changes. A subtle reduction of hair distribution was observed in physical examination. These characteristics described above were observed in cases of 46,XX testicular DSD published by Majzoub *et al*. (2017) in a literature review. The authors described the signs and symptoms of 46 men diagnosed with de la Chapelle Syndrome, which included sexual dysfunction, reduced hair distribution, and gynecomastia in 21% (4/19), 26.6% (8/30), and 40% (12/30) of the subjects. Less frequent abnormalities included undescended testicles (cryptorchidism) and hypospadias (Majzoub *et al*., 2017). Semen analysis was conducted after three days of abstinence. Decreased viscosity and azoospermia were observed in the samples, whereas the rest of the characteristics were within the parameters of normality (WHO, 2010). In addition, hormonal tests were performed using a chemiluminescence technique to analyze the patient's serum sample. Hormonal analyses revealed elevated levels of follicle stimulating hormone (FSH) and luteinizing hormone (LH). Free testosterone (FT) was below the expected level, while serum levels of prolactin and sex hormone binding globulin (SHBG) were within the normal range (Table 1). These results are typically seen in hypergonadotropic hypogonadism (HH) patients, thus showing that subjects with 46,XX testicular DSD usually develop HH (Baziz et al., 2016; Chen *et al*., 2019; Shi & Martin, 2000).

**Table 1.** Clinical and laboratory data of a patient with 46,XX testicular DSD.

Analyzed parameters	Data	Serum levels considerednormal for adult men
Age	38 years	
Height	183 cm	
External genitalia	Male	
Testicles	Atrophic	
FSH*	23.81 mUI/mL	0.95-11.95 mUI/mL
LH**	17.67 mUI/mL	0.57-12.07 mUI/mL
PRL***	8.7 ng/ml	2.1-17.7 ng/ml
SHBG****	31.8 nmol/L	13.2-89.5 nmol/L (age: 20-70)
FT*****	2.329 ng/dl	3.4-24.6 ng/dl (age: 17-40)

* Follicle stimulating hormone (FSH);

** luteinizing hormone (LH);

*** prolactin (PRL);

**** sex hormone-binding globulin (SHBG);

***** free testosterone (FT).

**Figure 1:**
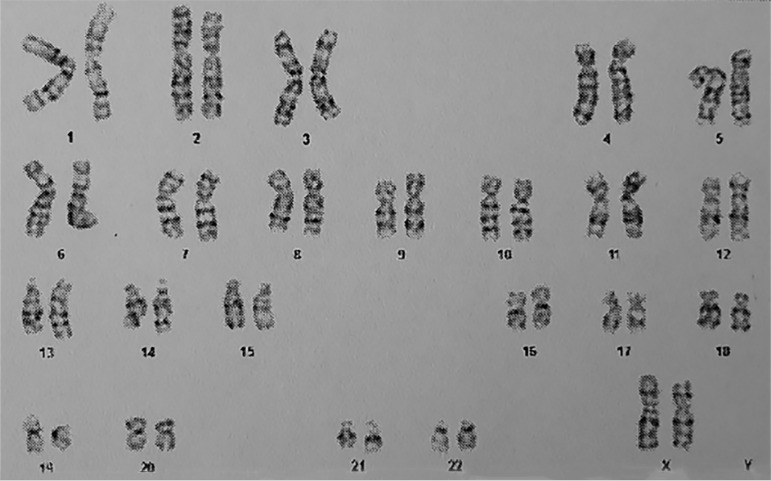
Chromosomal analysis by G-band karyotyping, showing chromosomal constitution 46,XX.

In addition to the results and reported characteristics, subjects with 46,XX testicular DSD exhibit testicular development in the absence of the Y chromosome (Bianco *et al*., 2011). Thus, peripheral blood G-band karyotyping was performed. A cytogenetic study revealed a 46,XX chromosomal constitution (Figure 1).

In reported sequences, the sex-determining region Y (SRY) gene is present in about 80% of individuals with de la Chapelle syndrome, as a result of the translocation of a fragment of the Y chromosome during parental spermatogenesis (Majzoub *et al*., 2017; Albu *et al*., 2019; Vorona *et al*., 2007). For the patient in question, real-time polymerase chain reaction analysis indicated the presence of the SRY gene. This gene is responsible for testicular development and regulation of the expression of genes such as SOX9 and DAX1, which play important roles in sexual determination, cell differentiation (Leydig cells, Sertoli cells, sperm), vascularization, and testicular cord development (Terribile *et al*., 2019; Barrionuevo & Scherer, 2010; Larney *et al*., 2014; She & Yang, 2017). Therefore, individuals with 46,XX testicular DSD possessing the SRY gene have male characteristics.

In situations where the SRY gene is not present in individuals with 46,XX testicular DSD, patients present varying degrees of masculinization. Mechanisms that might explain this phenotype include changes in genes related to sexual development (e.g., SOX9 and DAX1) or the activation of testicular differentiation cascades, occult Y-chromosome mosaicism limited to gonadal tissue or eliminated during development (Bianco *et al*., 2011; De La Chapelle, 1987; Croft et al., 2018; Bertalan *et al*., 2019; Rizvi, 2008).

When improperly investigated, it is difficult to diagnose 46,XX testicular DSD in phenotypically normal males. Identification of the condition can be performed during puberty, since approximately a third of the patients develop gynecomastia. However, individuals are often diagnosed with 46,XX testicular DSD male during infertility investigation, since all XX men are sterile (Majzoub *et al*., 2017; Terribile *et al*., 2019).

Once diagnosis was confirmed and the implications of having de la Chapelle syndrome were explained to the patient, a multidisciplinary approach consisting of genetic counseling, clinical management (hormone supplementation), psychological support, and referral for assisted reproductive technology treatments (in vitro fertilization or intrauterine insemination with donor semen) was offered to the couple. In addition, follow-up pelvic imaging to assess the presence of remaining Müllerian ducts (to avoid morbidities such as infections or urinary incontinence) and surgical removal of the gonads to avoid neoplastic transformation (gonadoblastoma) of dysgenetic gonads, since it may occur in about 30% of cases (Adrião *et al*., 2020), were recommended.

This paper reported a rare case of 46,XX testicular DSD diagnosed from the investigation of male factor infertility and emphasized the importance of producing an adequate diagnosis, performing thorough clinical and laboratory evaluation, and following up with assisted reproductive technology treatments with donor semen.
